# Changes in skeletal muscle and physical function after cardiac surgery: a prospective study

**DOI:** 10.1016/j.jnha.2026.100999

**Published:** 2026-10-03

**Authors:** Peter Svidt, Rikke Daugaard, Troels Lading, Ivy Susanne Modrau

**Affiliations:** a Dept. of Clinical Medicine, Aarhus University, Aarhus, Denmark; b Dept. of Cardiothoracic and Vascular Surgery, Aarhus University Hospital, Aarhus, Denmark; c Dept. of Cardiothoracic Surgery, Aalborg University Hospital, Denmark

**Keywords:** Sarcopenia, Body composition, Aged, Cardiac surgery, Computed tomography, Frailty, Myosteatosis

## Abstract

**Objectives:**

To assess postoperative changes in muscle mass and quality, factors associated with these changes, and sarcopenia prevalence in older cardiac surgery patients.

**Design:**

Secondary observational analysis of a randomized controlled early rehabilitation trial

**Setting:**

Tertiary University Hospital, Denmark.

**Participants:**

Patients aged ≥65 years undergoing cardiac surgery with available cardiac CT imaging (n = 100).

**Intervention:**

Not applicable.

**Measurements:**

Muscle quantity (skeletal muscle index, SMI) muscle quality (intermuscular adipose tissue-to-skeletal muscle area ratio, IMAT/SMCA), muscle strength (30-second chair stand test, 30CST), and physical performance (10-meter walking test, 10MWT) were assessed at baseline and 6 weeks after discharge. Sarcopenia was defined according to the revised European Working Group on Sarcopenia in Older People (EWGSOP2) criteria and compared with low muscle mass based on age- and sex-specific SMI reference cut-offs. Associations were examined using multivariable regression.

**Results:**

Among 100 patients (mean age 71.5 ± 4.4 years; 17% women), 74 had paired CT scans available. EWGSOP2-defined sarcopenia prevalence rose from baseline to 6 weeks (13.5%–21.6%), whereas SMI-defined low muscle mass was higher at both time points (18.9%–36.5%). SMI decreased by 7.1% (p < 0.001) and IMAT/SMCA increased by 14.7% (p < 0.001), with marked inter-individual variability. Preserved left ventricular ejection fraction (β = 2.33, 95% CI 0.14–4.52; p = 0.037) and allocation to early home-based rehabilitation (β = 1.34, 95% CI 0.01–2.68; p = 0.048) were associated with higher SMI. In supplementary analyses, higher EuroSCORE II was associated with poorer muscle quality (β = 0.037, 95% CI 0.018−0.056; p < 0.001). No consistent associations were observed between muscle measures and functional performance.

**Conclusion:**

Preoperative sarcopenia and early postoperative decline in muscle mass and quality were common in older cardiac surgery patients. CT identified muscle deterioration not fully captured by EWGSOP2 criteria, suggesting potential value for targeted preventive and rehabilitative interventions.

**Registration:**

ClinicalTrials.gov (NCT06370611).

## Highlights

• Sarcopenia is common and worsens after cardiac surgery in older adults
• Muscle mass and quality decline in most patients, but improve in some
• Higher disease burden is linked to postoperative decline in muscle quality
• Preserved left ventricular ejection fraction is associated with less muscle loss
• CT identifies low muscle mass missed by the EWGSOP2 functional criteria

## 1 Introduction

Older adults undergoing cardiac surgery frequently experience postoperative functional decline, prolonged recovery, and reduced quality of life [[1], [2], [3], [4], [5], [6]].

Sarcopenia, characterized by age-related loss of skeletal muscle mass and function, is increasingly recognized as a marker of vulnerability in older adults and is associated with frailty, impaired physical performance, mortality, and adverse outcomes after cardiac surgery [[7], [8], [9], [10], [11], [12], [13], [14]]. However, sarcopenia prevalence, perioperative skeletal muscle changes, and their relationship with postoperative functional outcomes remain insufficiently characterized in older adults undergoing cardiac surgery.

Computed tomography (CT) provides an objective assessment of skeletal muscle quantity, quality, and intermuscular adipose tissue [[15], [16], [17]]. CT-based body composition analysis is traditionally performed at the third lumbar vertebral level [18]. Muscle measurements at the eighth thoracic vertebral level (T8) correlate with whole-body muscle mass [19,20], enabling assessment of muscle health from routine preoperative cardiac CT without additional imaging or radiation exposure [21]. However, the clinical utility of opportunistic T8-based muscle assessment using routine preoperative cardiac CT in patients undergoing cardiac surgery has not been evaluated.

The aim of this study was to assess sarcopenia prevalence, perioperative changes in skeletal muscle quantity and quality, and associations with postoperative physical function in older adults undergoing cardiac surgery. We hypothesized that sarcopenia is common in this population and that postoperative decline in skeletal muscle mass and quality is associated with reduced physical function.

## 2 Methods

### 2.1 Study design and population

This prospective substudy was nested within the RECARD randomized trial investigating early rehabilitation after cardiac surgery in older adults [22]. Participants were randomized to 6-week mobile health-supported home-based rehabilitation in addition to standard care or standard care alone.

The substudy included participants from the sponsor site with preoperative or 6-week cardiac CT scans suitable for T8 muscle assessment and without intravenous contrast enhancement to ensure comparable attenuation measurements.

### 2.2 CT-derived body composition

CT images were analyzed at the T8 level using an artificial intelligence-assisted software platform (Data Analysis Facilitation Suite; DAFS, Voronoi Health Analytics, Vancouver, Canada) for automated tissue segmentation using predefined Hounsfield unit (HU) thresholds for muscle (−29 to 150 HU) and adipose tissue (−190 to −30 HU) [23]. Skeletal muscle index (SMI; cm^2^/m^2^) and the ratio of intermuscular adipose tissue to skeletal muscle cross-sectional area (IMAT/SMCA) were calculated as normalized measures of muscle quantity and quality, respectively. Low muscle quantity was defined using sex- and age-specific SMI cut-offs from a large community-based cohort [24].

### 2.3 Physical function and self-reported physical activity

All assessments were performed at baseline and 6-week follow-up. Physical function was assessed using the 30-second chair stand test (30CST) and 10-meter walk test (10MWT) [25,26].

Self-reported physical activity was assessed using two items from the Danish National Health Profile questionnaire capturing moderate and vigorous activity during a typical week [27]. Moderate-equivalent physical activity was calculated by weighting vigorous activity as twice the duration of moderate activity and classified according to WHO recommendations [28] as insufficient or sufficient (≥150 min/week).

### 2.4 Sarcopenia definition

Sarcopenia was classified according to EWGSOP2 criteria as probable, confirmed, or severe based on the 30-second chair stand test for muscle strength, CT-derived SMI for muscle quantity, and 10MWT gait speed for physical performance [9]. Age- and sex-specific reference values were used for muscle strength and muscle quantity [24,29], and gait speed <0.8 m/s defined low physical performance [9].

### 2.5 Statistical analysis

Continuous variables are presented as mean ± standard deviation or median (interquartile range), and categorical variables as counts and percentages. Analyses were restricted to participants with complete baseline and follow-up CT scans and functional assessments; no imputation was performed. Changes over time were calculated as follow-up minus baseline values.

Associations between CT-derived muscle parameters and physical function were assessed using correlation analyses.

Multivariable linear regression models using an ANCOVA approach were constructed with follow-up SMI and IMAT/SMCA as outcomes, adjusted for corresponding baseline values and predefined covariates (randomization group, age, body mass index, diabetes, renal function, left ventricular ejection fraction, and length of hospital stay). Sensitivity analyses replaced individual clinical covariates included in EuroSCORE II with log-transformed EuroSCORE II. Exploratory analyses evaluated self-reported physical activity as alternative exposure

### 2.6 Ethics approval and trial registration

The RECARD trial was approved by the Danish National Medical Research Ethics Committee (31 January 2024; no. 2314027) and the Central Denmark Region Internal Registry of Research Projects (30 January 2024; no. 1-16-02-33-24). The trial was registered at ClinicalTrials.gov (NCT06370611). The present substudy was conducted according to the trial protocol, with all participants providing written informed consent.

## 3 Results

### 3.1 Study population and CT-derived body composition

Of 100 included participants, CT-derived body composition data were available in 84 patients at baseline and 90 patients at 6-week follow-up; 74 had paired CT scans available for longitudinal analyses (Supplementary Fig. S1). Mean age was 71.5 ± 4.4 years, and 17% were women. Baseline characteristics are presented in Table 1 .

**Table 1:** Baseline characteristics of the study population overall and by treatment group.

Characteristic	Overall N = 100	Control N = 50	Intervention N = 50	SMD
Demographics				
Age (years)	71.5 ± 4.4	70.9 ± 4.5	72.2 ± 4.2	0.30
Male sex	83 (83%)	42 (84%)	41 (82%)	0.05
BMI (kg/m^2^)	27.0 ± 3.9	27.2 ± 4.5	26.8 ± 3.3	0.10
Current smoking	5 (5.0%)	3 (6.0%)	2 (4.0%)	0.09
Organ function measures				
NYHA class				0.36
I	27 (27%)	10 (20%)	17 (34%)	
II	59 (59%)	31 (62%)	28 (56%)	
III	14 (14%)	9 (18%)	5 (10%)	
LVEF %				
<50%	9 (9.0%)	5 (10%)	4 (8.0%)	0.07
Comorbidities				
Hyperlipidemia^a^	64 (64%)	33 (66%)	31 (62%)	0.08
Hypertension^a^	75 (75%)	36 (72%)	39 (78%)	0.14
Diabetes^a^	15 (15%)	9 (18%)	6 (12%)	0.17
COPD^a^	7 (7%)	2 (4%)	5 (10%)	0.24
PAD^b^	5 (5%)	3 (6%)	2 (4%)	0.09
Prior myocardial infarction	6 (6.0%)	3 (6.0%)	3 (6.0%)	0.00
Renal dysfunction^c^	14 (14%)	9 (18%)	5 (10%)	0.23
Operative characteristics				
Type of surgery				
CABG	46 (46%)	21 (42%)	25 (50%)	0.16
Off-pump CABG	4 (4.0%)	2 (4.0%)	2 (4.0%)	0.00
Valve surgery	60 (60%)	34 (68%)	26 (52%)	0.32
Other	1 (1.0%)	1 (2.0%)	0 (0%)	0.20
Extracorporeal circulation				
ECC time (min)	84 ± 37	89 ± 44	79 ± 30	0.28
Cross-clamp time (min)	58 ± 32	62 ± 36	53 ± 26	0.29
Risk Score				
EuroSCORE II	1.30 (0.87, 2.00)	1.60 (0.90, 2.10)	1.18 (0.87, 1.58)	0.13

Data are presented as mean ± SD, median (interquartile range), or number (%), as appropriate.

SMD, Standardized mean difference; BMI, body mass index; NYHA, New York Heart Association; LVEF, left ventricular ejection fraction; COPD, chronic obstructive pulmonary disease; PAD, peripheral arterial disease; eGFR, estimated glomerular filtration rate; CABG, coronary artery bypass grafting; ECC, extracorporeal circulation; EuroSCORE, European System for Cardiac Operative Risk Evaluation.

a Defined as pharmacological treatment for the respective condition.

b Defined according to EuroSCORE II.

c eGFR <60 mL/min/1.73 m^2^.

### 3.2 Sarcopenia and skeletal muscle changes

At 6-week follow-up, SMI decreased by 7.1% (P < 0.001), and IMAT/SMCA increased by 14.7% (P < 0.001), with substantial interindividual variability (Fig. 1). The prevalence of low SMI-defined muscle quantity increased from 18.9% to 36.5%, whereas EWGSOP2-defined sarcopenia increased from 13.5% to 21.6% (Fig. 2).

**Fig. 1:**
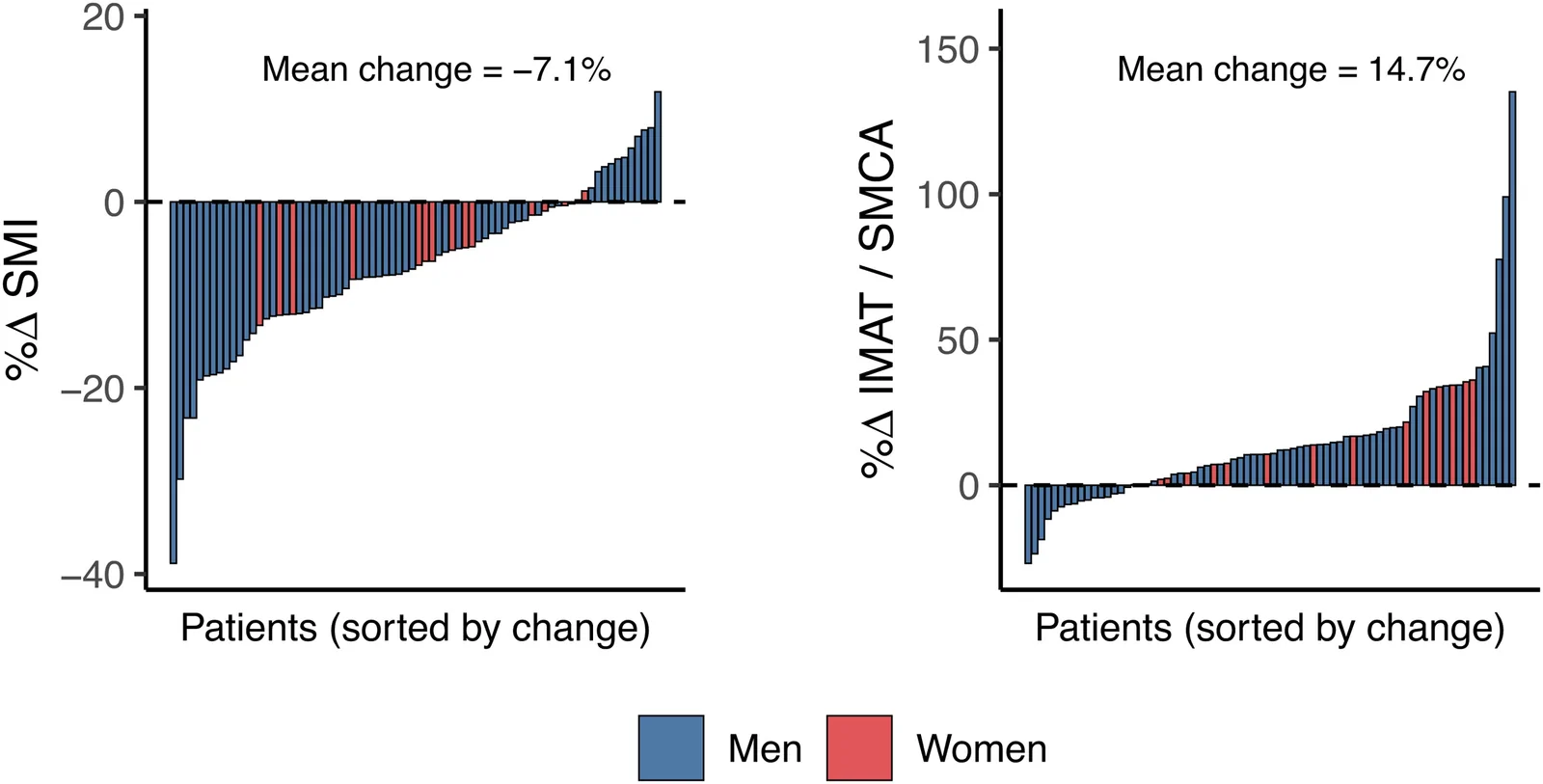
Individual postoperative changes in skeletal muscle mass and quality. Waterfall plots of individual percentage changes from baseline in skeletal muscle index (SMI) and IMAT-to-skeletal muscle area ratio (IMAT/SMCA) among 74 patients. Bars are ordered by magnitude of change; blue denotes men and red women. Mean changes are shown above each panel.

**Fig. 2:**
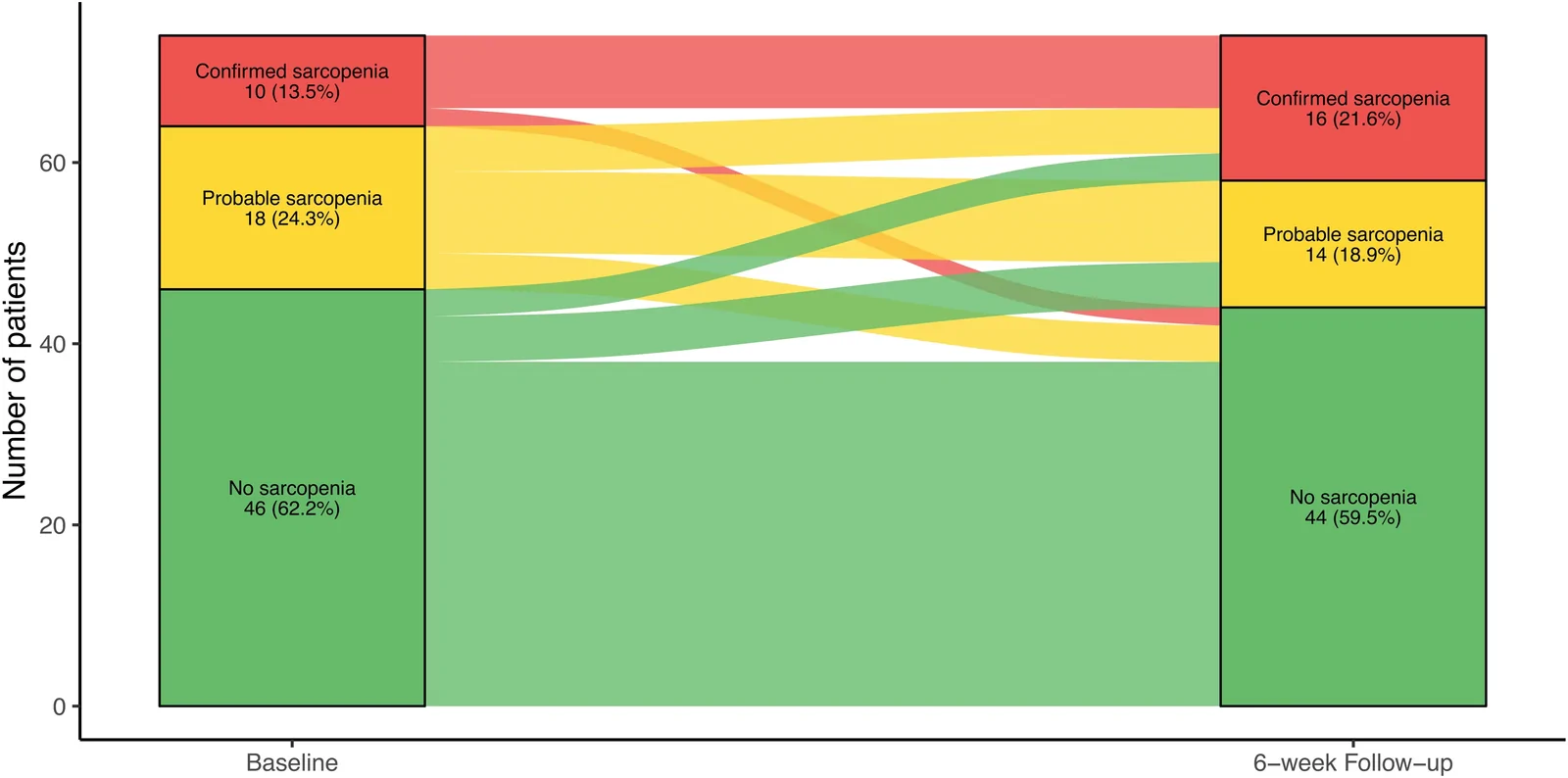
Progression of sarcopenia after cardiac surgery. Alluvial diagram of individual transitions in sarcopenia status from baseline to 6-week follow-up according to EWGSOP2 criteria. Colors indicate sarcopenia category (green = no sarcopenia; yellow = probable sarcopenia; red = confirmed sarcopenia).

No linear associations were observed between age and baseline CT-derived muscle parameters (Pearson r −0.19 to 0.41), with substantial interindividual variability across age groups.

### 3.3 Associations of CT-derived muscle parameters with physical function

No consistent associations were observed between CT-derived muscle parameters and physical function at baseline or follow-up. IMAT/SMCA showed the strongest associations, with moderate inverse correlations with 30-second chair stand test and 10-meter walk test performance at follow-up. Changes in CT-derived muscle parameters were not associated with changes in physical function. (Supplementary Fig. S2)

### 3.4 Associations of CT-derived muscle parameters with self-reported physical activity

At baseline, SMI and IMAT/SMCA did not differ between participants with self-reported physical activity below or above 150 min/week moderate-equivalent activity. At 6-week follow-up, IMAT/SMCA was lower among participants reporting activity above the WHO-recommended threshold, whereas SMI did not differ (Supplementary Figure S3). Randomization group allocation was similarly distributed across activity categories (P = 0.24). The distribution of randomization groups did not differ between physical activity categories (Fisher's exact test, p = 0.24).

### 3.5 Adjusted analyses

Results of multivariable regression analyses are presented in Supplementary Table S1.

Baseline muscle parameters were strongly associated with follow-up SMI and IMAT/SMCA in adjusted analyses (both p < 0.001).

For SMI, preserved left ventricular ejection fraction (≥50%) was associated with higher follow-up values (β = 2.33, 95% CI 0.14–4.52; p = 0.037), as was assignment to the intervention group compared with controls (β = 1.34, 95% CI 0.01–2.68; p = 0.048). Meeting the WHO physical activity recommendations was not associated with follow-up SMI (β = 0.26, 95% CI −1.27−1.79; p = 0.735).

For IMAT/SMCA, diabetes was associated with lower follow-up IMAT/SMCA (β = −0.041, 95% CI −0.074 to −0.007; p = 0.017). In supplementary analyses, higher EuroSCORE II was associated with higher follow-up IMAT/SMCA (β = 0.037, 95% CI 0.018−0.056; p < 0.001). Meeting the WHO physical activity recommendations was not associated with lower follow-up IMAT/SMCA (β = −0.018, 95% CI −0.042 to 0.006; p = 0.142).

## 4 Discussion

In this prospective substudy of a randomized trial, chest CT identified a substantial burden of low skeletal muscle mass and myosteatosis among older adults undergoing cardiac surgery, with further deterioration during the early postoperative period. Low SMI-defined muscle mass was more prevalent than EWGSOP2-defined sarcopenia both preoperatively and postoperatively. Changes in CT-derived muscle parameters were not consistently associated with changes in physical function or self-reported physical activity.

In our study, sarcopenia prevalence increased from 14% to 22%, whereas low SMI-defined muscle quantity increased from 19% to 37%, supporting the concept that the perioperative period represents a vulnerable phase for skeletal muscle deterioration [37]. The higher prevalence of low SMI-defined muscle quantity, together with the weak associations between CT-derived parameters and physical performance, indicates that structural and functional assessments provide complementary information.

CT-derived muscle assessment may therefore identify patients with impaired muscle composition who are not classified as having sarcopenia according to EWGSOP2 criteria.

In adjusted analyses, preserved left ventricular ejection fraction and assignment to early home-based rehabilitation were associated with higher follow-up SMI, whereas higher EuroSCORE II was associated with greater IMAT/SMCA. The association between rehabilitation assignment and SMI is consistent with a potential role of early mobilization on preservation of skeletal muscle, whereas associations with LVEF and EuroSCORE II may reflect differences in cardiovascular reserve and recovery capacity.

This study has several strengths, including its prospective design, standardized functional assessment, and AI-assisted CT-based evaluation of skeletal muscle composition with standardized analysis.

Several limitations should be considered. This single-center study included a modest sample size, which may limit generalizability. CT assessment at a single thoracic level may not fully represent whole-body muscle composition, although prior studies have shown reasonable correlations with volumetric measures. The short follow-up period, variability in rehabilitation adherence, and the use of self-reported physical activity as an exploratory measure may have influenced the observed associations. Longitudinal analyses were limited to participants with available paired CT scans, and selection bias cannot be excluded, as patients with greater frailty or postoperative complications may have been less likely to undergo follow-up imaging.

## 5 Conclusions

Cardiac surgery was associated with early deterioration in CT-derived muscle quantity and quality among older adults. CT-derived measures revealed a greater burden of muscle impairment than EWGSOP2-defined sarcopenia and may provide complementary information to identify patients with clinically relevant muscle deficits and inform rehabilitation strategies and clinical decision-making.

## CRediT authorship contribution statement

Peter Svidt: Data curation, formal analysis, visualization, writing - original draft,

Ivy Susanne Modrau: Conceptualization, Methodology, Writing - review & editing, Supervision, Funding acquisition.

Rikke Daugaard: Methodology, Data curation, Writing - review & editing, Project administration.

Troels Lading: Methodology, Writing – review & editing, Resources.

## Declaration of Generative AI and AI-assisted technologies in the writing process

During the preparation of this work, the authors used ChatGPT to assist with language editing. The authors reviewed and edited the content and take full responsibility for the final manuscript.

## Funding

This study was supported by an unrestricted grant from the Novo Nordisk Foundation (grant no. 0083480, 2023). The funder had no role in study design, data collection, analysis, interpretation, or manuscript preparation.

## Data statement

The data that support the findings of this study are available from the corresponding author upon reasonable request.

## Other information registration

The trial was registered at ClinicalTrials.gov (NCT06370611).

## Protocol

https://ugeskriftet.dk/dmj/early-remote-rehabilitation-improve-health-elderly-after-cardiac-surgery-study-protocol

## Declaration of competing interest

The authors declare that they have no known competing financial interests or personal relationships that could have appeared to influence the work reported in this paper.
