# Supplementary material for: Changes in skeletal muscle and physical function after cardiac surgery: a prospective study
Source: J Nutr Health Aging. 2026 Oct 3;30(12):100999. doi: 10.1016/j.jnha.2026.100999 (PMC13661222; doi:10.1016/j.jnha.2026.100999)
Supplement: Supplementary file 1 [file mmc1.pdf]

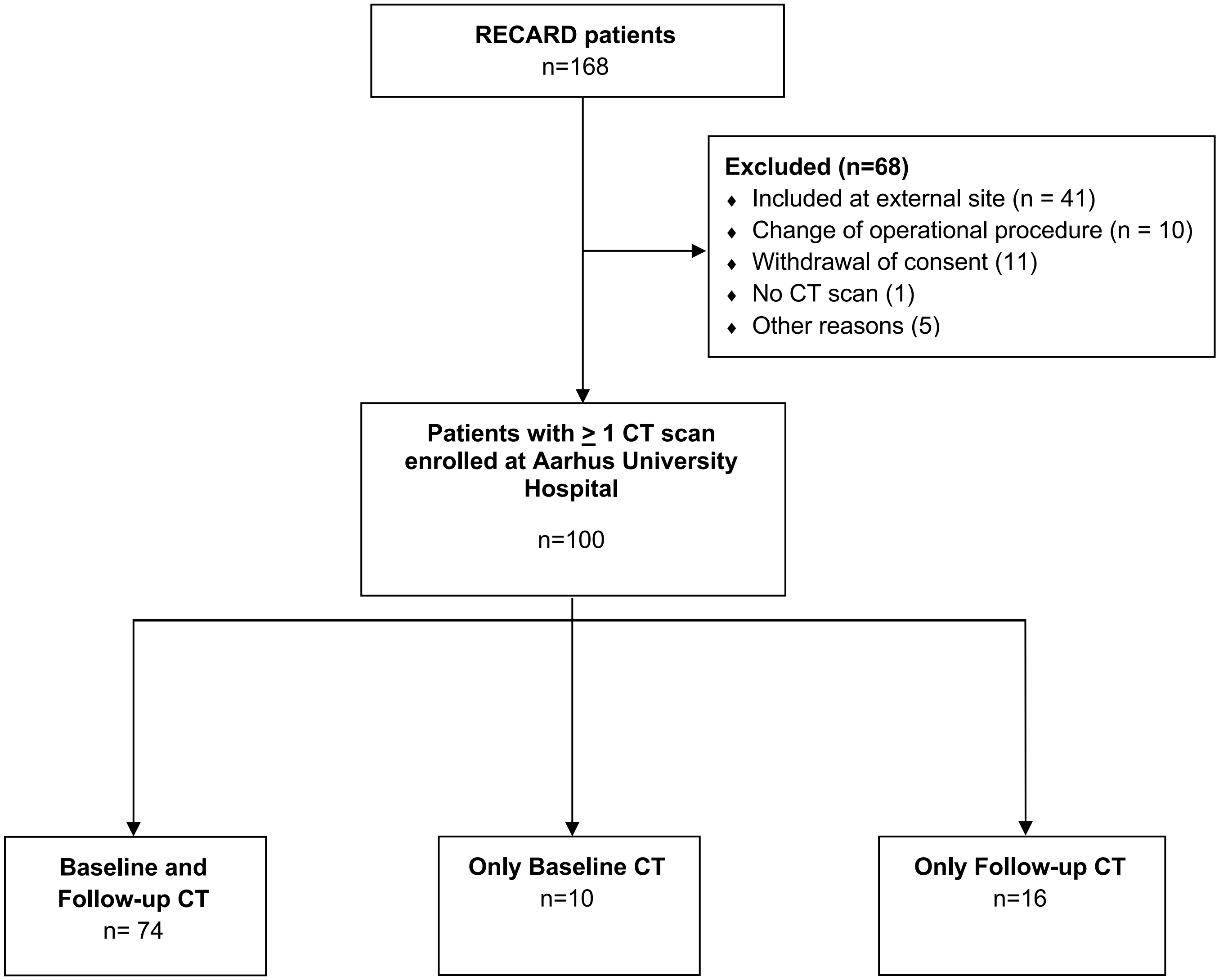

Participant flow chart
