# Supplementary material for: Changes in skeletal muscle and physical function after cardiac surgery: a prospective study
Source: J Nutr Health Aging. 2026 Oct 3;30(12):100999. doi: 10.1016/j.jnha.2026.100999 (PMC13661222; doi:10.1016/j.jnha.2026.100999)
Supplement: Supplementary file 2 [file mmc2.pdf]

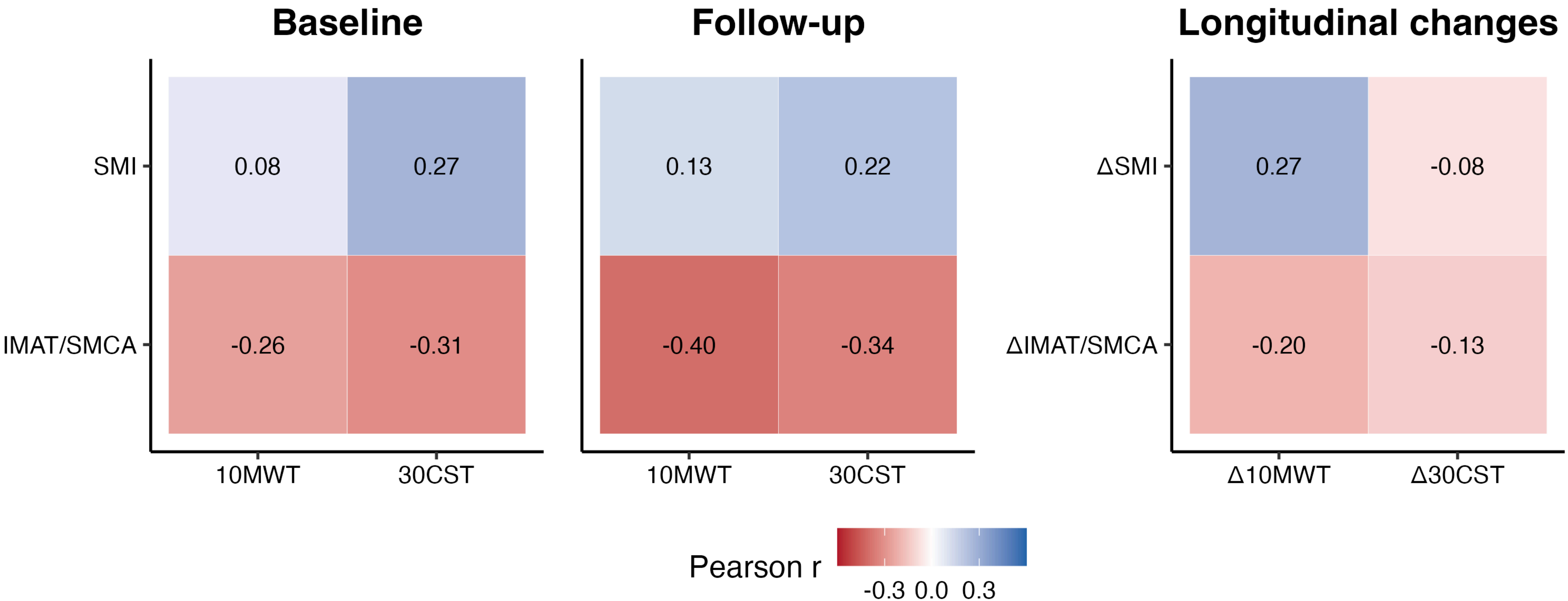
Baseline
Follow-up
Longitudinal changes
SMI
IMAT/SMCA
0.08
0.27
-0.26
-0.31
10MWT
30CST
0.13
0.22
-0.40
-0.34
10MWT
30CST
ΔSMI
ΔIMAT/SMCA
0.27
-0.08
-0.20
-0.13
Δ10MWT
Δ30CST
Pearson r
-0.3 0.0 0.3
