# Supplementary material for: Changes in skeletal muscle and physical function after cardiac surgery: a prospective study
Source: J Nutr Health Aging. 2026 Oct 3;30(12):100999. doi: 10.1016/j.jnha.2026.100999 (PMC13661222; doi:10.1016/j.jnha.2026.100999)
Supplement: Supplementary file 3 [file mmc3.pdf]

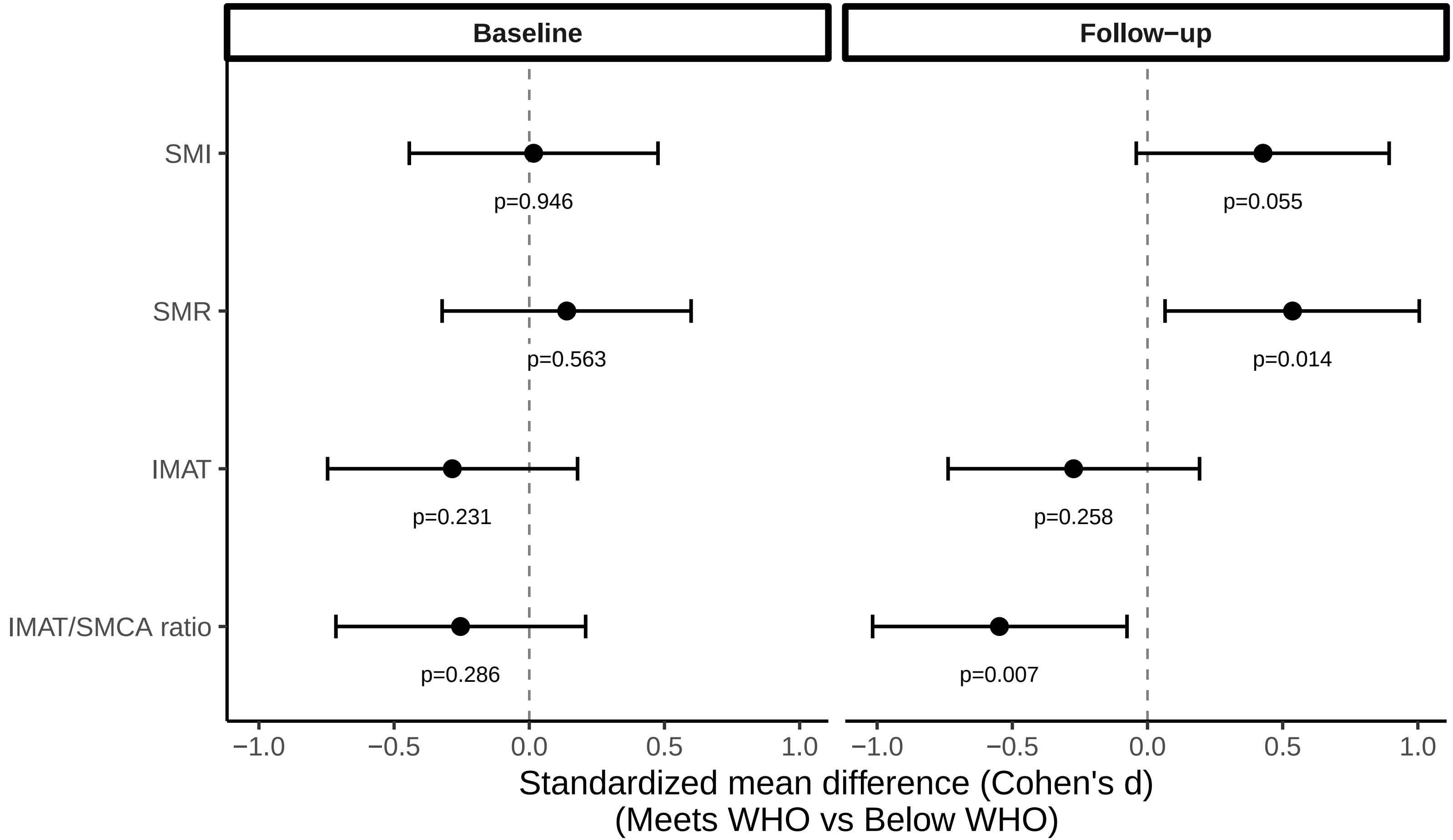
Baseline
Follow−up
SMI
SMR
IMAT
IMAT/SMCA ratio
p=0.946
p=0.563
p=0.231
p=0.286
p=0.055
p=0.014
p=0.258
p=0.007
−1.0
−0.5
0.0
0.5
1.0
Standardized mean difference (Cohen's d)
(Meets WHO vs Below WHO)
