# Supplementary material for: Changes in skeletal muscle and physical function after cardiac surgery: a prospective study
Source: J Nutr Health Aging. 2026 Oct 3;30(12):100999. doi: 10.1016/j.jnha.2026.100999 (PMC13661222; doi:10.1016/j.jnha.2026.100999)
Supplement: Supplementary file 4 [file mmc4.docx]

## Supplementary Table 1. Multivariable linear regression analyses of follow-up muscle outcomes

| **Panel A. Primary analyses** | | | | | | |
| --- | --- | --- | --- | --- | --- | --- |
| **Variable** | **SMI β (95% CI)** | **p-value** | **Variable** | **IMAT/SMCA β (95% CI)** | | **p-value** |
| Baseline SMI | 0.80 (0.70 to 0.90) | <0.001 | Baseline IMAT/SMCA | 1.01 (0.90 to 1.13) | | <0.001 |
| Intervention group | 1.34 (0.01 to 2.68) | 0.048 | Intervention group | -0.007 (-0.029 to 0.015) | | 0.545 |
| Age | 0.01 (-0.16 to 0.18) | 0.899 | Age | 0.001 (-0.002 to 0.003) | | 0.649 |
| BMI | -0.06 (-0.23 to 0.10) | 0.458 | BMI | 0.001 (-0.002 to 0.005) | | 0.410 |
| Diabetes | 1.21 (-0.82 to 3.23) | 0.237 | Diabetes | -0.041 (-0.074 to -0.007) | | 0.017 |
| LOS ≥7 days | -0.34 (-1.69 to 1.00) | 0.611 | LOS ≥7 days | -0.006 (-0.028 to 0.016) | | 0.602 |
| eGFR ≥60 | -0.36 (-2.54 to 1.82) | 0.742 | eGFR ≥60 | -0.013 (-0.049 to 0.022) | | 0.458 |
| LVEF ≥50% | 2.33 (0.14 to 4.52) | 0.037 | LVEF ≥50% | -0.004 (-0.040 to 0.032) | | 0.825 |
| **Panel B. Sensitivity analyses** | | | | | | |
| **Variable** | **SMI β (95% CI)** | **p-value** | **Variable** | **IMAT/SMCA β (95% CI)** | **p-value** | |
| Baseline SMI | 0.78 (0.68 to 0.88) | <0.001 | Baseline IMAT/SMCA | 0.978 (0.873 to 1.084) | <0.001 | |
| Intervention group | 1.17 (-0.08 to 2.42) | 0.067 | Intervention group | -0.001 (-0.020 to 0.019) | 0.952 | |
| BMI | -0.05 (-0.21 to 0.11) | 0.540 | BMI | 0.002 (-0.001 to 0.005) | 0.183 | |
| LOS ≥7 days | -0.09 (-1.39 to 1.20) | 0.889 | LOS ≥7 days | -0.008 (-0.028 to 0.012) | 0.409 | |
| Log EuroSCORE II | -1.25 (-2.55 to 0.05) | 0.058 | Log EuroSCORE II | 0.037 (0.018 to 0.056) | <0.001 | |
| **Panel C. Exploratory analyses** | | | | | | |
| **Variable** | **SMI β (95% CI)** | **p-value** | **Variable** | **IMAT/SMCA β (95% CI)** | **p-value** | |
| Baseline SMI | 0.79 (0.68 to 0.90) | <0.001 | Baseline IMAT/SMCA | 0.995 (0.870 to 1.120) | <0.001 | |
| WHO PA | 0.26 (-1.27 to 1.79) | 0.735 | WHO PA | -0.018 (-0.042 to 0.006) | 0.142 | |
| Age | 0.05 (-0.12 to 0.23) | 0.549 | Age | 0.001 (-0.002 to 0.003) | 0.662 | |
| BMI | -0.05 (-0.24 to 0.13) | 0.572 | BMI | 0.002 (-0.002 to 0.005) | 0.285 | |
| Diabetes | 0.95 (-1.26 to 3.15) | 0.394 | Diabetes | -0.040 (-0.074 to -0.006) | 0.022 | |
| LOS ≥7 days | -0.43 (-1.93 to 1.07) | 0.568 | LOS ≥7 days | -0.008 (-0.031 to 0.016) | 0.520 | |
| eGFR ≥60 | 0.19 (-2.19 to 2.57) | 0.875 | eGFR ≥60 | -0.016 (-0.053 to 0.021) | 0.386 | |
| LVEF ≥50% | 2.51 (0.22 to 4.80) | 0.032 | LVEF ≥50% | -0.006 (-0.043 to 0.030) | 0.733 | |

Multivariable linear regression analyses of follow-up muscle outcomes Data are β coefficients (95% CI). Panel A presents the primary multivariable models, and Panel B presents sensitivity analyses including EuroSCORE II. SMI, skeletal muscle index; IMAT, intramuscular adipose tissue; SMCA, skeletal muscle cross-sectional area; BMI, body mass index; eGFR, estimated glomerular filtration rate; LVEF, left ventricular ejection fraction; EuroSCORE II, European System for Cardiac Operative Risk Evaluation II; WHO PA, meeting WHO physical activity recommendations; CI, confidence interval.
